# Selenium and viral infection: are there lessons for COVID-19?

**DOI:** 10.1017/S0007114520003128

**Published:** 2020-08-06

**Authors:** Giovanna Bermano, Catherine Méplan, Derry K. Mercer, John E. Hesketh

**Affiliations:** 1 Centre for Obesity Research and Education (CORE), School of Pharmacy and Life Sciences, Robert Gordon University, Aberdeen AB10 7GJ, UK; 2 School of Biomedical, Nutritional and Sport Sciences, Faculty of Medical Sciences, Newcastle University, Newcastle upon Tyne NE2 4HH, UK

**Keywords:** Selenium, COVID-19, Inflammation, Viral infection, Severe acute respiratory syndrome coronavirus 2, Redox status, Micronutrients, COVID-19, coronavirus disease 2019, CVB, coxsackievirus B3, ER, endoplasmic reticulum, GPX, glutathione peroxidase, IFN, interferon, IV, influenza virus, MERS, Middle East respiratory syndrome, NF-κB, nuclear factor kappa-light-chain-enhancer of activated B cells, NOX, NADPH oxidase, Nrf2, nuclear factor erythroid 2-related factor 2, ROS, reactive oxygen species, SARS-CoV-2, severe acute respiratory syndrome coronavirus 2, SELENOF, selenoprotein F, SELENOS, selenoprotein S, TXNRD, thioredoxin reductase, UPR, unfolded protein response

## Abstract

Se is a micronutrient essential for human health. Sub-optimal Se status is common, occurring in a significant proportion of the population across the world including parts of Europe and China. Human and animal studies have shown that Se status is a key determinant of the host response to viral infections. In this review, we address the question whether Se intake is a factor in determining the severity of response to coronavirus disease 2019 (COVID-19). Emphasis is placed on epidemiological and animal studies which suggest that Se affects host response to RNA viruses and on the molecular mechanisms by which Se and selenoproteins modulate the inter-linked redox homeostasis, stress response and inflammatory response. Together these studies indicate that Se status is an important factor in determining the host response to viral infections. Therefore, we conclude that Se status is likely to influence human response to the severe acute respiratory syndrome coronavirus 2 (SARS-CoV-2) infection and that Se status is one (of several) risk factors which may impact on the outcome of SARS-CoV-2 infection, particularly in populations where Se intake is sub-optimal or low. We suggest the use of appropriate markers to assess the Se status of COVID-19 patients and possible supplementation may be beneficial in limiting the severity of symptoms, especially in countries where Se status is regarded as sub-optimal.

A link between Se deficiency and human disease was first demonstrated by the discovery in China that the aetiology of Keshan disease involved both coxsackievirus infection and a low intake of the micronutrient Se^(1,2)^. Since those initial observations, several studies have investigated how Se intake affects responses to viral infections^(3)^ and the importance of understanding these links is highlighted by the coronavirus disease 2019 (COVID-19) pandemic^(4)^. This is of particular relevance because, although severe Se deficiency is rare, there is considerable evidence that sub-optimal Se status is common with a significant proportion of the population across the world potentially in this category^(5)^. Fish and shellfish are relatively high in Se content but, for most foods, Se content is dependent on the level of Se present in the soil; this geographical variation is reflected in the Se status of different human populations, many of which have blood Se that is regarded as sub-optimal (≤85 µg/l)^(5,6)^, for example, in parts of Europe including the UK.

Severe acute respiratory syndrome coronavirus 2 (SARS-CoV-2) is the infectious agent responsible for the current COVID-19 pandemic. It is a coronavirus with a positive-sense single-stranded RNA genome (26–32 kb), one of the largest of the RNA viruses^(7,8)^. Other clinically important RNA viruses include those responsible for respiratory diseases, such as severe acute respiratory syndrome (SARS), Middle East respiratory syndrome (MERS) and influenza, and for other infectious diseases such as poliomyelitis and HIV infection^(3)^. SARS-CoV-2 targets cells expressing the angiotensin-converting enzyme 2 receptor; these include airway epithelial cells, alveolar epithelial cells, vascular endothelial cells and macrophages in the lung as well as myocardial and renal cells^(9,10)^. SARS-CoV-2 infection is characterised by a highly aggressive inflammatory response. Pyroptosis of infected lung epithelial cells, as a result of viral replication, triggers an inflammatory cascade leading to the release of pro-inflammatory cytokines and chemokines^(9)^. The cytokine storm (hypercytokinaemia) observed in severe cases induces widespread damage to the lung epithelium that can facilitate the development of secondary bacterial or fungal infections^(11)^. In addition to the local damage, the cytokine storm and uncontrolled inflammation affect other parts of the body, in particular the myocardial and renal systems, and can lead to septic shock, multi-organ failure and death^(12,13)^.

The COVID-19 pandemic raises three major questions in relation to Se: (i) to what extent does Se intake, particularly sub-optimal intake as well as severe deficiency, affect responses to viral infection; (ii) what are the biochemical mechanisms supporting a role of Se in the host response and (iii) could Se intake be a factor in determining the response to COVID-19 infection? In this article, we address these questions by firstly reviewing the literature concerning the effects of Se on RNA virus infections, secondly by exploring how Se modulates the inter-linked redox homeostasis, stress response and inflammatory response mechanisms and finally by considering these effects in relation to recent observations on COVID-19.

## Host selenium status and viral infection

The first evidence that there might be a link between Se status and susceptibility of humans to a viral infection came from the study of Keshan disease, a myocardiopathy associated with heart failure and death, among the population in Heilongjiang province in China^(1)^. The Se content of food and drinking water was found to be extremely low in areas where the disease was endemic. Addition of Se-containing fertiliser to the soil and direct nutritional supplementation led to a large reduction in the incidence of the acute form of the disease^(1,14)^. However, the seasonal variation of the disease suggested additional contributing factors, and postmortem samples from patients were found to contain the coxsackievirus B3 (CVB) virus, a single-stranded RNA virus. It is now clear that Keshan disease is due to a combination of infection with CVB and low Se status. In mice, CVB can lead to myocarditis, inflammation of the heart. The virulence of CVB strains varies and crucially it was observed that a non-virulent strain, which caused no disease in animals fed a Se-adequate diet, caused severe myocarditis in animals fed a Se-deficient diet^(14)^. In addition, sequencing of the viral genome revealed that passage through Se-deficient mice led to mutations in the viral genetic sequence and increased pathogenicity^(15,16)^. Similarly, an influenza virus (IV) strain causing mild pneumonitis induced much greater pathogenicity in Se-deficient mice^(17,18)^. Furthermore, as with CVB, Se status also affected IV mutation, with low Se status promoting rapid evolution in haemagglutinin (*HA*) and neuraminidase (*NA*) genes^(19)^. An *in vitro* study on bronchial epithelial cells showed that Se supplementation lowered the extent of apoptotic cell death after infection with IV^(18)^.

Few data are available concerning the relationship between Se status and HIV susceptibility or disease progression^(3)^. However, two studies indicate that sub-optimal Se status lowers the number of CD4 T cells and increases both disease progression and death rates in HIV-infected patients^(20,21)^. Se deficiency is commonly observed in HIV-infected patients and has been linked to increased mortality. In a study of 125 HIV-1 seropositive drug-using men and women, CD4 T cell counts over time and Se deficiency (but not deficiency of vitamin A, vitamin B_12_ or Zn) were significantly associated with mortality, indicating that Se deficiency is an independent predictor of survival for those with HIV-1 infection^(21)^.

The incidence of haemorrhagic fever with renal syndrome, a public health problem in China caused by RNA hantaviruses, is about six times higher in severely Se-deficient areas of China compared with non-Se-deficient areas, and negatively correlated with the Se content of crops and feeds^(22)^. Surprisingly, plasma concentration and activity of the selenoprotein glutathione peroxidase 3 (GPX3) were significantly higher in haemorrhagic fever with renal syndrome patients compared with controls, but this may indicate increased inflammation rather than differences in Se status. Finally, *in vitro* Se supplementation (200 ng/ml) significantly lowered viral copy number in human umbilical vein endothelial cells (HUVEC) infected with hantavirus at a low multiplicity of infection^(22)^. Although no human studies have assessed the impact of Se status on the disease associated with West Nile virus, another RNA virus, *in vitro* infection of kidney epithelial cells leads to lower virus-induced cell death in Se-supplemented conditions^(23)^.

The effects of Se supplementation on viral infection in humans were studied in UK volunteers of marginal Se status (plasma Se < 95 µg/l) who were given an attenuated polio virus. Se supplementation was found to both increase the rate at which the virus was cleared from the body and decrease the rate at which mutations appeared in the viral genome^(24)^. In addition, measurements of immune markers indicated that supplemental Se accelerated the cellular antiviral response. Importantly, this study not only supports animal work on IV and CVB showing that higher Se status enhanced the host response to a viral infection and promoted generation of mutations that increase virulence of RNA viruses, but also provides evidence that similar effects occur in humans within a range of Se status found commonly in the UK population and worldwide.

In summary, there is strong evidence that low Se status in both animals and humans influences the host response to a number of RNA viruses with low Se status leading to more severe forms of the disease. Furthermore, in CVB and IV infections, low Se status also promotes generation of mutations and increased virulence. The impact of Se status on the ability of a host to respond to viral infection is reflected in the crucial role of Se and selenoproteins in cellular and molecular mechanisms involved in the control of redox homeostasis, stress response, immune and inflammatory response.

## Selenoproteins, cell stress response mechanisms and viral infection

**Fig. 1.**
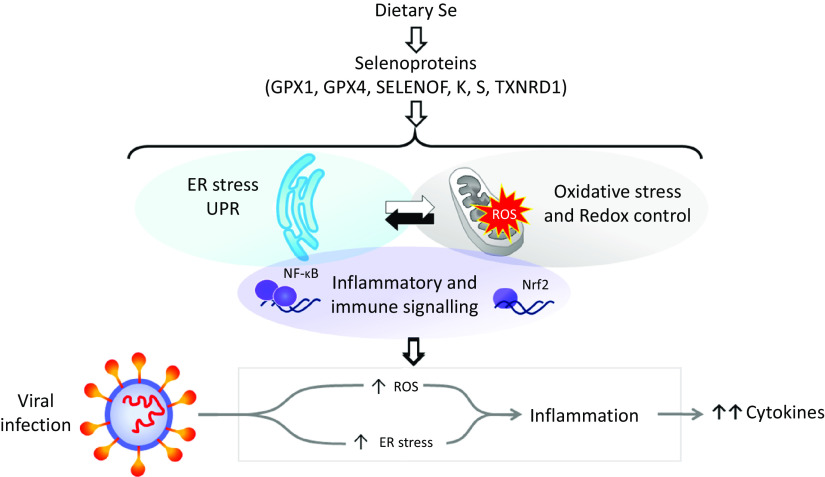
Selenium and response to viral infection. In humans, dietary selenium is incorporated into twenty-five selenoproteins as the amino acid selenocysteine. Selenoproteins include glutathione peroxidases (GPX), 15 kDa selenoprotein F (SELENOF), selenoproteins K and S and thioredoxin reductases (TXNRD). They play crucial roles in molecular pathways such as oxidative stress response, redox control and mitochondrial function, as well as endoplasmic reticulum (ER) stress and unfolded protein responses (UPR), and immune and inflammatory response in particular involving NF-*κ*B and nuclear factor erythroid 2-related factor 2 (Nrf2) signalling. Cross-talk between these pathways has been shown to be key to produce an adequate response to viral infections, and low selenium status has been linked to poorer response to RNA virus infection in human studies, and animal and cell culture models. The diagram illustrates how reduced expression of selenoproteins as a result of low/sub-optimal selenium status could alter molecular pathways involved in stress responses and contribute to an aggressive pro-inflammatory environment leading to poorer disease prognosis.

Dietary Se is converted in the body into the amino-acid selenocysteine which is then incorporated into proteins, selenoproteins, during their synthesis by a unique mechanism which involves a specific tRNA for selenocysteine and recoding of a UGA stop codon as codon for selenocysteine^(25,26)^. To date, twenty-five genes coding for selenoproteins have been identified. The biological activity of Se is carried out primarily through its presence in these selenoproteins, the functions of which centre around roles in antioxidant protection, redox homeostasis and endoplasmic reticulum (ER) stress^(27)^ (Fig. 1). Thus, GPX1–4 have well-documented, important roles in the metabolism of, and protection from, reactive oxygen species (ROS) including peroxides and lipid hydroperoxides. Thioredoxin reductases (TXNRD) function in redox homeostasis and signalling, and a series of less well-characterised selenoproteins including selenoprotein F, K, M, N and S (SELENOF, SELENOK, SELENOM, SELENON and SELENOS) are present in the ER where they play roles in protein folding and protection from oxidative stress. In both human and mouse models, transcriptomic and proteomic analyses have indicated that, in colonic tissue and leucocytes, the major downstream target pathways affected by sub-optimal Se intake are immune response and inflammatory signalling, nuclear factor kappa-light-chain-enhancer of activated B cells (NF-*κ*B) signalling and ER stress response pathways^(28–30)^. Such changes are consistent with the known roles of selenoproteins and highlight that changes in Se intake can affect the pathways that determine cell responses to oxidative and ER stress as well as the immune response.

### Oxidative stress response and redox homeostasis

Viral-induced oxidative stress through generation of ROS is crucial to the viral lifecycle and its pathogenicity^(31)^. Se, through the activity of antioxidant selenoproteins, has the potential to enhance the response to viral-induced oxidative stress and to contribute to the maintenance of redox homeostasis. Se supplementation, which increases GPX1 expression and lowers oxidative stress in a cell culture model, reduces the replication rate of porcine circovirus 2, a DNA virus^(32)^. Data from GM animal models (knock-out) show that, contrary to wild-type mice, animals lacking GPX1 developed myocarditis when infected by a benign CVB strain, in a similar manner to mice fed a Se-deficient diet^(33)^. Similarly, following IV infection, GPX1 knock-out mice displayed an increased and prolonged tissue inflammation, higher levels of macrophages in bronchial fluid and higher levels of the pro-inflammatory cytokine TNF-*α*; furthermore, some effects were reversed by ebselen (2-phenyl-1,2-benzisoselenazol-3(2 H)-one), a synthetic organoselenium compound/drug with GPX-mimetic properties^(34)^. Lower selenoprotein levels in mice expressing a mutant selenocysteine tRNA gene were found to alter the response to IV infection, with increased levels of lung pro-inflammatory cytokines and interferon-*γ* (IFN-*γ*) and slow viral clearance rates, although there was no effect on lung pathology^(35)^. Since these mice showed only a small 20 % reduction in GPX1 expression, it is possible that there is a threshold activity of GPX1 and other selenoproteins above which there is little effect on virus infection. Overall, these data suggest that GPX1 is important for appropriate cell and tissue responses to a viral infection, in particular in relation to the inflammatory response to respiratory viruses.

Changes in redox homeostasis, disruption of antioxidant defences, chronically elevated levels of ROS and induction of ROS-generating enzymes, such as NADPH oxidase (NOX) and xanthine oxidase, are key events linked to infection with respiratory viruses, including IV and SARS-CoV^(31,36)^. Increased levels of ROS and reactive nitrogen species have been reported in lung tissues of patients who died in fatal IV pandemics, while studies with IV-infected mice and cell lines also demonstrated an enhanced production of ROS and disturbance of antioxidant defence^(37–39)^. Suppression of chronic oxidative stress by targeting pro-oxidant enzymes such as NOX and dual oxidase (DUOX) could, therefore, ameliorate host response to viral infection. At present, no evidence is available for a role of Se or selenoproteins in modulating NOX and DUOX activity in relation to viral infections; however, supplementation with selenomethionine has been shown to protect lung tissue against toxic effects of ionising radiation by reducing *DUOX1* and *2* gene expression and IL-4 and IL-4 receptor subunit *α*-1 protein levels^(40)^. Moreover, GPX and TXNRD have been shown, in endothelial cells, to play an important role in controlling the metabolism and physiological function of ROS and reactive nitrogen species, whose production is regulated by NOX and xanthine oxidase^(41)^. Control of ROS levels associated with endothelial damage and inflammation may provide a potential mechanism by which Se status modulates the host response to viral infection, and this could be significant in the case of SARS-CoV-2 infection as both endothelial damage and inflammation have been linked to COVID-19 aetiology^(42)^.

As a first line of defence, epithelial barriers often undergo significant damage as a result of the release/production of ROS. Through their antioxidant functions, selenoenzymes such as GPX protect epithelial barriers from ROS by reducing lipid and phospholipid hydroperoxides, and thus, adequate Se status could also contribute to the maintenance of the structural and functional integrity of respiratory epithelial barriers. Similarly, antioxidant selenoproteins protect neutrophils from endogenous oxidative stress^(43)^.

Mitochondrial dysfunction has also been linked to viral infection. In IV infection, mitochondria are an important source of ROS due to the virus-induced leakage of electrons from the respiratory chain^(44)^. Similarly, a study by Shi *et al*.^(45)^ reported that a protein encoded by SARS-CoV, designated as open reading frame-9b (ORF-9b), localised to mitochondria and caused mitochondrial elongation, resulting in evasion of host innate immunity. Although to date there is no evidence that during respiratory virus infection the alteration of mitochondrial function is caused by lower selenoprotein expression, a considerable amount of data indicates that selenoproteins such as GPX4, GPX1 and TXNRD1 are crucial to maintain mitochondrial function and redox homeostasis^(46,47)^. Thus, low Se status has the potential to affect response to respiratory viruses by impacting on the expression of selenoproteins key to mitochondrial function (Fig. 1).

### Endoplasmic reticulum stress

The ER is important for protein synthesis, folding, processing and post-translational modifications. During viral infections, the protein load exceeds the ER folding and processing capacity, and activation of various signalling pathways, collectively known as ER stress response or unfolded protein response (UPR), occurs as part of the host antiviral response^(48)^. ER redox state, ER stress and Ca^2+^ signalling are tightly regulated by a complex antioxidant system, which includes seven ER-resident selenoproteins – 15 kDa selenoprotein (SELENOF), type 2 iodothyronine deiodinase (DIO2) and SELENOS, N, K, M and T (Fig. 1). As a group, they are implicated in a range of processes including ER stress and inflammation (reviewed in Addinsall *et al*.^(49)^). Cross-talks between ER function, NF-*κ*B signalling and mitochondrial function^(50)^ during immune response and antiviral defence highlight that these mechanisms are inter-linked (Fig. 1). More specifically, in Z *α*(1)-antitrypsin (ZAAT) deficiency, a disease associated with emphysematous lung disease, SELENOS was found to affect ER function and NF-*κ*B signalling, and this effect was enhanced following Se supplementation^(51)^. In addition, silencing of SELENOS in vascular smooth muscle cells, enhanced markers of ER stress suggesting that SELENOS might protect cells by inhibiting oxidative and ER stress^(52)^. Mice with targeted deletion of SELENOK exhibited decreased viral clearance and elevated viral titres in the brain upon West Nile virus infection. Interestingly, these mice showed decreased Ca^2+^ flux in the ER of T cells, macrophages and neutrophils and lower T cell proliferation suggesting a role for SELENOK in immune response^(53)^. In human volunteers, SELENOS expression in peripheral blood mononuclear cells was found to increase after an IV challenge, and furthermore this increase was enhanced after Se supplementation^(54)^.

These findings, although not all closely related to respiratory viral infections, demonstrate several roles for selenoproteins in regulating ER stress and suggest they are important in determining ER stress and inflammatory responses during viral infections. SARS-CoV accessory viral protein affects several UPR components, suggesting that such viruses may directly modulate ER stress responses^(55)^. Since ER stress and inflammation are inter-linked^(56)^, it is likely that UPR induction may play a significant role in the host response to SARS-CoV-2. Furthermore, there is a close relationship between ER stress, oxidative stress and inflammation. ER stress can lead to inflammation and conversely inflammation can induce UPR and ER stress^(56)^. High production of free radicals by immune cells, especially macrophages, at the site of infection, triggers oxidative stress. Excessive extracellular ROS/reactive nitrogen species initiates signalling cascades that lead to the onset of the inflammatory response, generating inflammatory mediators such as IL-1*β*, IL-6 and TNF-*α* and activating NF-*κ*B signalling pathways^(57–60)^.

## Selenium, inflammation, immune response and viral infections

As described in the above sections, there is extensive evidence suggesting that the inter-linked biochemical pathways of oxidative stress, ER stress and inflammation are induced during viral infection. This is true for a number of respiratory viruses and the limited data concerning SARS-CoV and SARS-CoV-2 indicate that they affect these pathways. Lung cell lines infected with either SARS-CoV-2, IV or MERS-CoV showed that up-regulation of antiviral IFN signalling was observed with all three viral infections, whereas up-regulation of the cytokine/inflammatory processes, down-regulation of mitochondrial organisation and respiration processes, and perturbation in the autophagic processes were specifically observed in SARS-CoV-2-infected cells and were absent in IV-infected cells^(61)^. These data highlight that, in SARS-CoV-2 infection, both mitochondrial function and inflammatory signalling were affected, which in turn could affect the immune response and lead to severe outcomes in the host.

In infections caused by SARS-CoV or SARS-CoV-2, pronounced inflammation and increased secretion of IL-1*β*, IL-4, IL-10, IFN-*γ*, IFN-*γ*-induced protein 10 (IP-10) and monocyte chemoattractant protein 1 (MCP-1) were observed^(4,62)^. In addition, production of several NF-*κ*B-mediated cytokines, including IL-6 and IL-8, has been detected in human bronchial epithelial cells in response to SARS-CoV infection^(63)^, whereas patients with severe COVID-19 in intensive care units exhibit elevated plasma levels of several cytokines (IL-2, IL-7, IL-10, TNF-*α*, MCP-1, granulocyte-colony stimulating factor (GCSF), IP-10 and macrophage inflammatory proteins (MIP1A)), suggesting a cytokine storm, resulting in hyper-inflammation^(64)^. Furthermore, evidence exists for IV strains to activate the nuclear factor erythroid 2-related factor 2/antioxidant response element (Nrf2/ARE) defence pathway, *in vitro* and in mice, subsequently leading to the activation of the NF-*κ*B pathway and cytokine production^(65,66)^, suggesting a potential important role for the Nrf2-NF-*κ*B cross-talk in associated pathogenesis, even if further evidence is required for other respiratory viruses.

Moderate Se deficiency was found to activate Nrf2 and the Wnt pathways which may have negative effects on protection against oxidative stress and inflammation in cancer cell models^(67)^; in addition, TXNRD1 has been shown to be a potent regulator of Nrf2 activation in lung epithelial cells^(68)^. Using a combined transcriptomics and proteomics approach to analyse gene and protein expression in rectal biopsies of healthy individuals, sub-optimal Se status was found to be associated with inhibition of NF-*κ*B, IL-1*β*, IL-6 and TNF-*α* signalling and down-regulation of immune and inflammatory response pathways^(30)^. Similar effects were observed in the colon of mice fed a mildly deficient Se diet^(28)^. In mice, a genomic approach combined with cytokine measurements has shown that a low Se diet led to increased activity of IFN-*γ* and IL-6 pathways as well as IL-6 levels^(69)^. Furthermore, following a lipopolysaccharide challenge, IL-6 levels were found to be greater in mice fed a low Se diet^(70)^, while, in women, lower Se status was associated with higher plasma IL-6 levels^(71)^. In addition, uncontrolled inflammation is associated with an altered balance of pro-inflammatory and anti-inflammatory eicosanoids. Animal studies have shown that increased Se intake leads to a rebalance towards more anti-inflammatory eicosanoids^(72)^, probably through modulation of lipoxygenase and cyclooxygenase activities as a result of increased GPX activity lowering levels of hydroperoxides and lipid radicals and so altering peroxide tone. Increased Se intake also causes lower expression of pro-inflammatory NF-*κ*B signalling^(73)^. There is also evidence for epigenetic regulation of inflammatory gene expression by Se^(74)^. Overall, Se intake affects a range of inflammatory mechanisms including Nrf2, eicosanoid and NF-*κ*B signalling pathways^(30,67,68,72,73)^ and therefore, individuals with sub-optimal/low Se status may exhibit an inadequate inflammatory response to respiratory RNA viral infections.

Alongside inflammation, SARS-CoV-2 infection triggers a local immune response, recruiting macrophages and monocytes that respond to the infection, release cytokines and prime adaptive T and B cell immune responses^(9)^. In particular, the immune response induced by SARS-CoV-2 infection is two phased: an initial phase in which specific adaptive immune responses are required to eliminate the virus and to preclude disease progression to the second and more severe phase that is characterised by severe lung damage (e.g. acute respiratory distress syndrome) and in which suppression of inflammation may be beneficial^(75)^. Therefore, strategies to boost immune responses are certainly important as, in some cases, a dysfunctional immune response occurs, which can cause severe lung and even systemic pathology.

Adequate Se levels have been shown to be required for the differentiation and proliferation of a number of immune cells involved in innate and adaptive immunity. In particular, at moderate doses, Se supplementation increases T cell proliferation and natural killer cell activity^(76,77)^. Moreover, Se supplementation of individuals with marginally low plasma levels increased cellular immune response to live polio vaccine and virus clearance^(24)^; although Se supplementation did not directly affect antibody titre after flu vaccination^(78)^, supplementation of individuals with marginally low Se status (92–98 µg/l) resulted in some beneficial effect on cellular immunity, with significantly higher T cell proliferation in the group supplemented with 100 μg/d selenomethionine in yeast matrix^(78)^. However, higher supplementation doses (200 μg/d selenomethionine in yeast matrix) led to a reduction in granzyme positive CD8 cells, and other forms of Se (e.g. Se-onions) did not impact on the immune response to flu vaccination^(78)^. These data complement the review by Steinbrenner and colleagues^(79)^ and suggest that optimising plasma Se status may improve immune responses, but high supplemental doses may have detrimental effects. Finally, as part of the innate immune response to pathogens, the methionine sulfoxide reductase B1 (MSRB1) selenoenzyme has been found, in murine macrophages, to regulate, through a redox mechanism and remodelling of the actin cytoskeleton, macrophage activation and phagocytosis^(80)^ and to promote anti-inflammatory cytokine production; the ablation of the *MSRB1* gene induced instead excessive pro-inflammatory cytokine production^(81)^.

## Selenium status and COVID-19

**Fig. 2.**
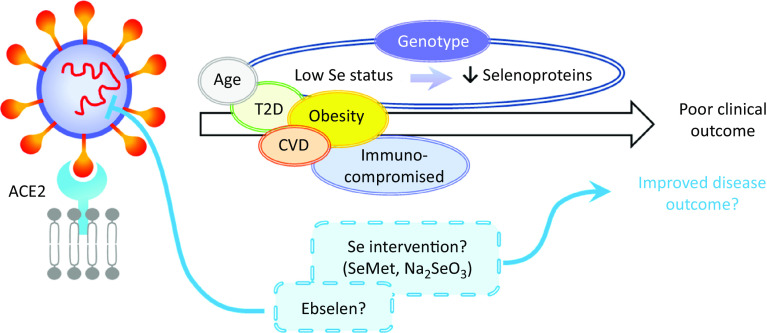
Hypothetical model of the impact of selenium on severe acute respiratory syndrome coronavirus 2 (SARS-CoV-2) infection. Disease outcome of a coronavirus disease 2019 (COVID-19) patient is influenced by age, obesity and the presence of co-morbidities such as type 2 diabetes (T2D), CVD and by being immunocompromised. Based on the current knowledge of the effects of selenium status on RNA virus infections, it can be speculated that patients with low/sub-optimal selenium status and reduced selenoprotein expression could be at higher risk of poor disease outcome. This could be influenced not only by dietary intake but also by genotype for a number of genetic variants in selenoprotein genes. Nutritional intervention such as selenium supplementation with selenomethionine (SeMet) or sodium selenite (Na_2_SeO_3_) may therefore improve disease outcomes for individuals with sub-optimal selenium status. Furthermore, therapeutic interventions may also include synthetic selenium compounds such as ebselen that has been found to inhibit SARS-CoV-2 main protease (M^pro^). ACE2, angiotensin-converting enzyme 2.

The available data suggest that several respiratory viruses induce changes in redox, ER stress and inflammation pathways that may be modulated by Se or selenoproteins. It appears that SARS-CoV-2 may induce comparable changes in these pathways but, to date, it is not known whether low Se status modulates the effect of the virus on such pathways and so enhances the cytokine storm observed in severe cases of COVID-19. Future studies should address whether this is the case, using both relevant animal and cell models and by assessing Se status and plasma inflammatory markers of patients with severe *v*. mild forms. Blood levels of SELENOP, the major form of Se in blood, are lowered by inflammation making the use of appropriate markers of Se status such as hair Se or erythrocyte Se important in human studies^(82,83)^. Such studies could inform the potential use of Se supplementation to limit the inflammatory response of patients (Fig. 2).

It is widely accepted that SARS-CoV-2 originated in Wuhan, Hubei province in China and rapidly spread across Hubei province and, subsequently, globally. Prior to the emergence of SARS-CoV-2, the population of Hubei was reported to show a wide range of hair Se content (0·06–0·79 mg/kg), albeit with a small sample size^(84)^. This wide range is compatible with both Se deficiency perhaps not being uncommon in Hubei province and there also being known high-Se areas. Interestingly, a recent ecological analysis using these data in relation to the COVID-19 outbreak in China indicates that there may be a relationship between Se status and disease outcome^(85)^. Cure and death rates from COVID-19 were analysed in different regions in relation to Se status as previously assessed by hair Se; notably, the city of Enshi in Hubei province, known for high Se status, showed a significantly higher cure rate, whereas Heilongjiang province, known to be an area of low Se status, showed a higher death rate. Overall, cure rate was associated with higher Se status. Although the association described in the present study is not based directly on Se status in COVID-19 patients, the work highlights that further, more detailed studies of Se status, COVID-19 infection and disease outcomes are vitally important. In addition, since levels of Se reported in some of the areas relevant to the present study are higher than those needed to optimise selenoprotein activities such future studies should address the range of Se intake effective at giving any protection.

Worst disease outcomes and higher mortality for COVID-19 patients have been associated with age, as well as other morbidities including obesity, type 2 diabetes and cardiovascular disorders that are often characterised by malnutrition^(86)^ and immune-compromised individuals (Fig. 2). Low levels, or intakes, of micronutrients including Se, Zn and vitamins A, B_6_, B_12_ and E have been linked to adverse outcomes in viral infection^(87)^ and levels of these micronutrients, as well as vitamins C and D, *n*-3 PUFA and iron should be considered in COVID-19 patients^(88)^. Obesity and high BMI have been found to be associated with severe forms of COVID-19^(89,90)^, possibly due to obesity-induced chronic inflammation. Potential explanations for these findings could include poor nutritional status of obese individuals, pre-existing impaired immune response or amplified pro-inflammatory responses and/or high affinity of SARS-CoV-2 for angiotensin-converting enzyme 2 (ACE2) receptor, highly expressed in adipose tissue^(91,92)^. Similarly, obesity has been shown to increase the duration of IV shedding and to be an independent risk factor for hospitalisation and death in H1N1 influenza^(93,94)^, and it has been suggested that the adipose tissue could represent a reservoir for SARS-CoV-2, IV and HIV^(89,95)^.

Furthermore, several studies indicate that Se levels are lower in obese individuals and a recent systematic review confirmed both that mean levels of Se in blood/serum were lower in the obese compared with healthy adult subjects and that mean blood GPX activity was decreased in obese individuals^(96)^. Interestingly, the authors reported that Se levels were higher in subjects with the metabolic syndrome.

Se derivative compounds have also been used as therapeutic agents. In particular, the organoselenium compound ebselen exhibits antioxidant properties, as well as anti-inflammatory, and anti-bacterial and antiviral properties^(97)^. Recently, using a combination of virtual and high-throughput technologies to screen 10 000 compounds, ebselen was identified among the most efficient potential inhibitor against COVID-19 main protease (M^pro^) and demonstrated strong antiviral activity in cell-based assays^(98,99)^. As clinical trials have shown ebselen can be safely used in humans^(100,101)^, this compound represents an interesting potential therapeutic candidate for COVID-19 (Fig. 2).

Given the strong evidence that Se protects against viral infection and supports an adequate immune response, it seems that individuals with low Se status could benefit from supplemental Se to prevent the development of severe forms of COVID-19. However, considering the ongoing debate around the risk-benefit window at an individual level^(26)^, Se supplementation should be restricted to individuals with low or sub-optimal Se intake/status to avoid toxicity associated with high Se intake. Since a number of SNP in genes encoding selenoproteins have been found to have functional consequences for Se metabolism and risk for a number of multifactorial diseases^(26)^, it is possible that genetic factors may also influence any effect of Se status on viral infection progression. As regards selenoproteins’ importance in redox homeostasis or ER stress response, a variant in the *SELENOS* gene has been reported to affect levels of inflammatory cytokines^(102)^, one in *GPX4* to affect monocyte adhesion to endothelial cells^(103)^ and others to affect inflammatory disease and cancer risk. It is interesting to speculate that such genetic variations, in combination with Se status, may impact on the ability of an individual to produce an adequate immune response to, and combat oxidative stress that occurs during viral infection (Fig. 2). Although to date no SNP in a Se pathway have been linked to altered response to infectious pathogens, other genetic variants in key host immune response genes in the host immune system have been shown to modulate both disease outcome and response to vaccination^(104)^.

An additional link, albeit novel and speculative, between SARS-CoV-2 and Se metabolism is through the mechanism of nonsense-mediated RNA decay. Coronaviruses, of which SARS-CoV-2 is one, have been reported to interfere with nonsense-mediated RNA decay^(105)^, a cellular mechanism that is also thought to play a role in regulating the pattern of synthesis of selenoproteins^(106,107)^. It can, therefore, be envisaged that SARS-CoV-2 infection may alter selenoprotein metabolism and thus the effects of Se on various downstream functions: an effect that would be exacerbated if Se intake is low. Future research should investigate this relationship.

## Conclusions

A better understanding of the immune and inflammatory responses that occur during COVID-19 is essential to develop adequate therapeutic approaches. Clearly, there are many factors that influence the response to SARS-CoV-2 infection and the subsequent disease outcomes. Given the crucial role of nutrition, and in particular micronutrients such as Se, in the immune and inflammatory responses^(88,108–110)^, it is also fundamental to understand how nutritional status could influence the ability to respond to the SARS-CoV-2. The significant body of evidence from human, animal and cell culture studies indicates that Se status is an important factor in response to viral infections, notably RNA viral infections and including respiratory virus infections. Understanding of these effects is strengthened by knowledge of the underlying biochemical mechanisms discussed in this review and together indicate that Se status may also influence human response to the SARS-CoV2 infection. The one limited study to date suggests that Se status is indeed associated with COVID-19 disease outcome^(85)^ and, therefore, we propose that Se status is an additional risk factor that should be considered as a determinant of SARS-CoV2 infection outcome, particularly in populations where Se intake is sub-optimal or low. Susceptibility to SARS-CoV2 infection is dependent on many factors such as age, BMI, obesity, type 2 diabetes and cardiovascular dysfunction, and in this context of COVID-19 progression being multifactorial, the relative importance of Se status remains to be determined. However, we suggest that the use of appropriate markers such as erythrocyte Se^(83)^ to monitor Se status of COVID-19 patients and possible supplementation may be beneficial in limiting the severity of symptoms, and secondly that authorities in countries where Se status is regarded as sub-optimal may consider Se supplementation as a public health strategy. Finally, on the basis of findings with other RNA viruses, it will be important to assess the extent to which Se status affects the mutation rate and pathogenicity of SARS-CoV-2.
